# Estrous Sheep Serum‐Derived Extracellular Vesicles Deliver Proteins That Modulate Sperm Motility in Sheep

**DOI:** 10.1111/rda.70293

**Published:** 2026-09-30

**Authors:** Clara Vidal‐Garcia, Marta Neila‐Montero, Eva Díaz‐Hernández, Paula Bóveda, Carmen María Picazo‐Córdoba, Vidal Montoro, Olga García‐Álvarez, José Julián Garde, Ana Josefa Soler, María Iniesta‐Cuerda

**Affiliations:** ^1^ Grupo SaBio IREC (UCLM‐CSIC‐JCCM), ETSIAMB Albacete Spain; ^2^ ETSIA, Ciudad Real (UCLM) Ciudad Real Spain

**Keywords:** ESS, EVs‐proteome, kinematic parameters, small ruminant species

## Abstract

Sperm capacitation requires signals from the female reproductive environment, although the molecular mechanisms governing this process remain poorly understood. Estrous sheep serum (ESS) is widely used in in vitro fertilization of small ruminants, contributing to mimic the female reproductive fluids. Among ESS components, extracellular vesicles (ESS‐EVs) may mediate the transfer of molecular signals to spermatozoa and support the modifications observed when sperm are incubated with ESS. To explore this possibility, ESS‐EVs were isolated and validated according to MISEV2023 guidelines, and their proteome was characterized and compared with the protein signature of sperm incubated with ESS. In addition, sperm were co‐incubated with ESS‐EVs, and sperm motility and mitochondrial activity were assessed. Notably, 27.1% of proteins previously reported to be upregulated in capacitated ram sperm were identified in ESS‐EVs, representing a significant enrichment of capacitation‐related factors. Functionally, ESS‐EVs modulated sperm kinematics, producing a motility profile comparable to that observed under capacitation conditions, while mitochondrial activity remained unchanged. Together, these findings suggest that ESS‐EVs may act as molecular conveyors delivering proteins that reshape sperm motility.

## 1 Introduction

The acquisition of fertilizing ability by mammalian sperm is a complex and tightly regulated process that must occur before they can fertilize the oocyte. Immediately after ejaculation, sperm cells are not yet fertilization‐competent and must undergo a series of physiological and biochemical modifications within the female reproductive tract, a process known as capacitation. Under experimental conditions, capacitation can be induced in vitro by incubating sperm in media designed to mimic the environment of the female reproductive tract. In small ruminant species, estrous sheep serum (ESS) is necessary for in vitro fertilization (García‐Álvarez et al. 2015), and it has been described that ESS promotes several canonical features of capacitation, including hyperactivated motility and the increase of protein tyrosine phosphorylation (Peris‐Frau et al. 2020).

Recent proteomic analyses in ovine sperm have shown that the incubation of sperm with ESS is associated with the upregulation of 280 proteins (Chen et al. 2024), raising the question of how such molecular components are acquired during this process, particularly given that spermatozoa are translationally inactive. A mechanistically plausible explanation is that ESS exerts part of its effect through the transfer of proteins packaged within extracellular vesicles (EVs). Unlike freely dissolved proteins, which may be prone to degradation in solution, EVs provide a protected system for delivering molecular cargo to spermatozoa. Indeed, spermatozoa are capable of interacting with and incorporating EVs, thereby acquiring their cargo (Armani et al. 2026). EV‐mediated communication could therefore emerge as a mechanism through which signals required to prime sperm for fertilization are transferred to sperm cells. Here, we characterized the protein cargo of ESS‐derived EVs (ESS‐EVs) and compared it with the signature of sperm incubated with ESS. In addition, sperm were co‐incubated with ESS‐EVs to evaluate their impact on sperm physiology.

## 2 Materials and Methods

### 2.1 Experimental Design

To evaluate whether ESS‐EVs may contribute to the molecular signature acquired by sperm after ESS incubation, EVs were first isolated from ESS and their protein cargo was characterized by proteomic analysis. The resulting ESS‐EVs proteome was then compared with a dataset of 280 proteins previously reported to be upregulated in ram sperm after incubation with ESS (Table S1; Chen et al. 2024). Moreover, ESS‐EVs were incubated with sperm to evaluate functional effects on sperm physiology (Figure 1).

**FIGURE 1:**
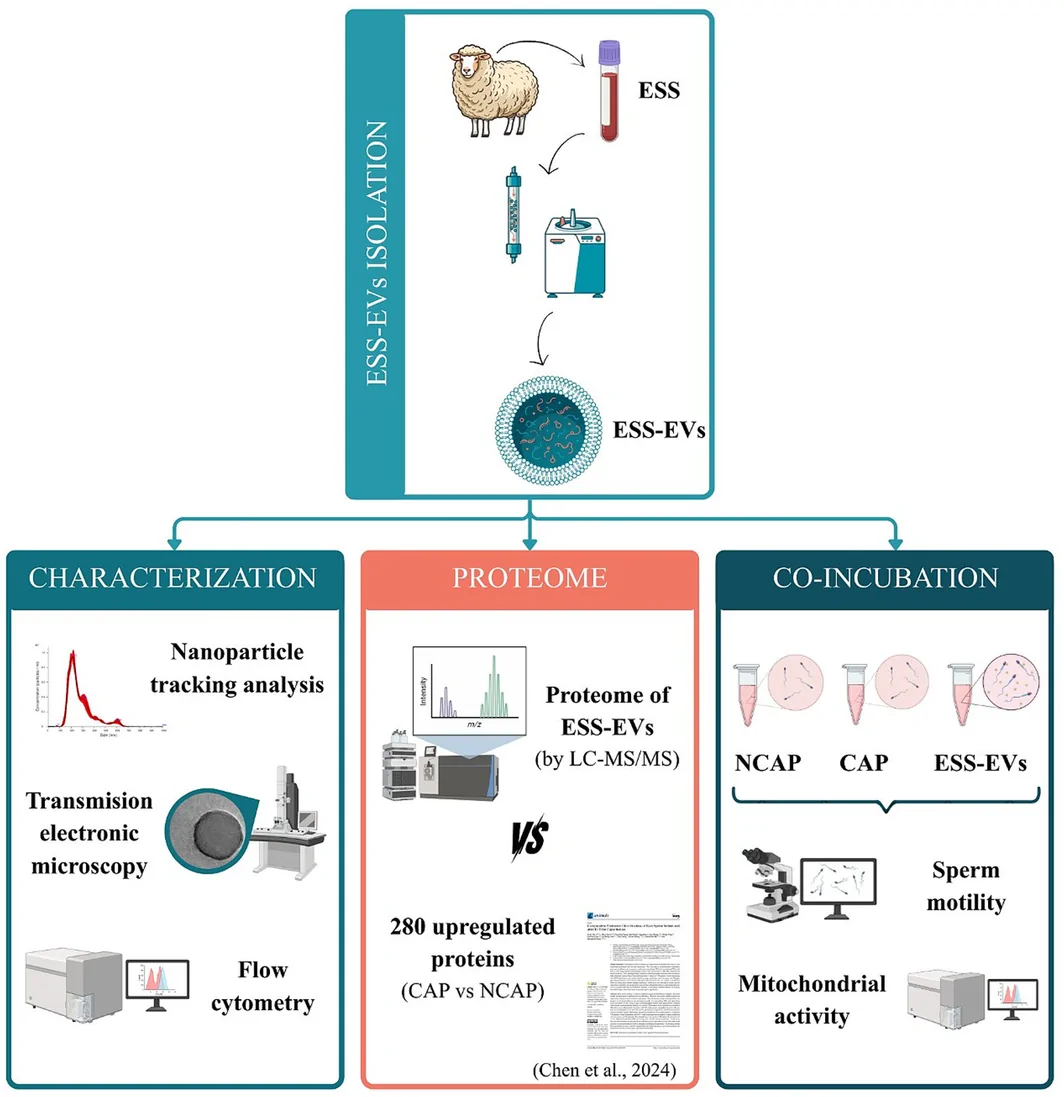
Experimental design for the comparison of estrus sheep serum (ESS) extracellular vesicles (ESS‐EVs) proteome with that of capacitated samples, and their effects on sperm function. Positive and negative controls were included adding ESS (2%; CAP) or not (NCAP) to a synthetic oviductal fluid.

### 2.2 EV Isolation and Characterization

EV isolation and characterization were done following MISEV2023 guidelines (Welsh et al. 2024). Serum from estrous ewes was processed for EV isolation by size‐exclusion chromatography and concentrated by ultracentrifugation. EVs were characterized by nanoparticle tracking analysis (NTA), transmission electron microscopy (TEM), and flow cytometry detecting CD9, CD63, and CD81 (Cajas et al. 2025). EV purity was further verified by the assessment of non‐EV markers BSA and calnexin as previously described (Diaz‐Muñoz et al. 2026).

### 2.3 Proteomic Analysis

Proteomic analysis was performed on three independent biological replicates, each consisting of an ESS‐EV pool generated from the estrous serum of six different donor ewes (18 donors in total). Samples were analysed at the Proteomics Facility of SCSIE, University of Valencia (ProteoRed member) using LC–MS/MS (timsTOF SCP, dia‐PASEF). Data processing and quantification were performed with DIA‐NN v1.8 within the FragPipe pipeline using an in silico predicted UniProt library (uniprotkb_ovis_205_03_28.predicted.speclib). The raw proteomics data have been deposited in the ProteomeXchange Consortium via the PRIDE partner repository and are available under the dataset identifier PXD080237.

### 2.4 Sperm‐EVs Coincubation and Functional Analysis

Sperm depleted of seminal plasma via percoll gradients as described by García‐Álvarez et al. (2015) were obtained from three rams (ETSIAMB farm, with authorization of Regional Government [ES008007]) and co‐incubated with ESS‐EVs (10 × 10^6^ sperm/mL; 100 × 10^6^ EVs/mL in a synthetic oviductal fluid (SOF); 1 h–38.5°C). Positive and negative controls were included adding ESS (2%; CAP) or not to the SOF (NCAP). Then, motility and mitochondrial activity were assessed by computer‐assisted analysis and flow cytometry. Although the complete set of CASA kinematic parameters was recorded, progressive sperm motility (SM), straight‐line velocity (VSL), linearity (LIN), and amplitude of lateral head displacement (ALH) were selected as representative descriptors of sperm motility. Mitochondrial activity was assessed in the viable sperm population using MitoTracker Deep Red FM, following the flow cytometry protocol previously described (Peris‐Frau et al. 2020).

### 2.5 Bioinformatics and Statistical Analysis

Bioinformatics and statistical analysis were performed in R. Proteins detected in ≥ 2 of 3 ESS replicates were retained and intersected with those proteins that were described as upregulated by Chen et al. (2024). Overlap significance was assessed by hypergeometric test (background: 22,024 *Ovis aries* proteins), and functional enrichment was performed using DAVID (*p* < 0.05). Comparisons between NCAP, CAP, and ESS‐EVs were assessed using a one‐way ANOVA with Tukey post hoc test (*n* = 4).

## 3 Results

The results of NTA evaluation for the validation of ESS‐EVs isolation revealed a mean size of 214.17 ± 13.07 nm and a concentration of 2.19 × 10^11^ ± 1.31 × 10^11^ particles/mL (Figure 2A). TEM confirmed typical spherical morphology and lipid bilayer structures (Figure 2B). Flow cytometry confirmed CFSE positivity (96.35% ± 4.74%) and the expression of canonical tetraspanins CD9 (17.84% ± 1.77%), CD63 (35.52% ± 8.13%), and CD81 (22.8% ± 6.27%, Figure 2C). Additionally, the absence of negative markers BSA and calnexin was verified (Figure 2D).

**FIGURE 2:**
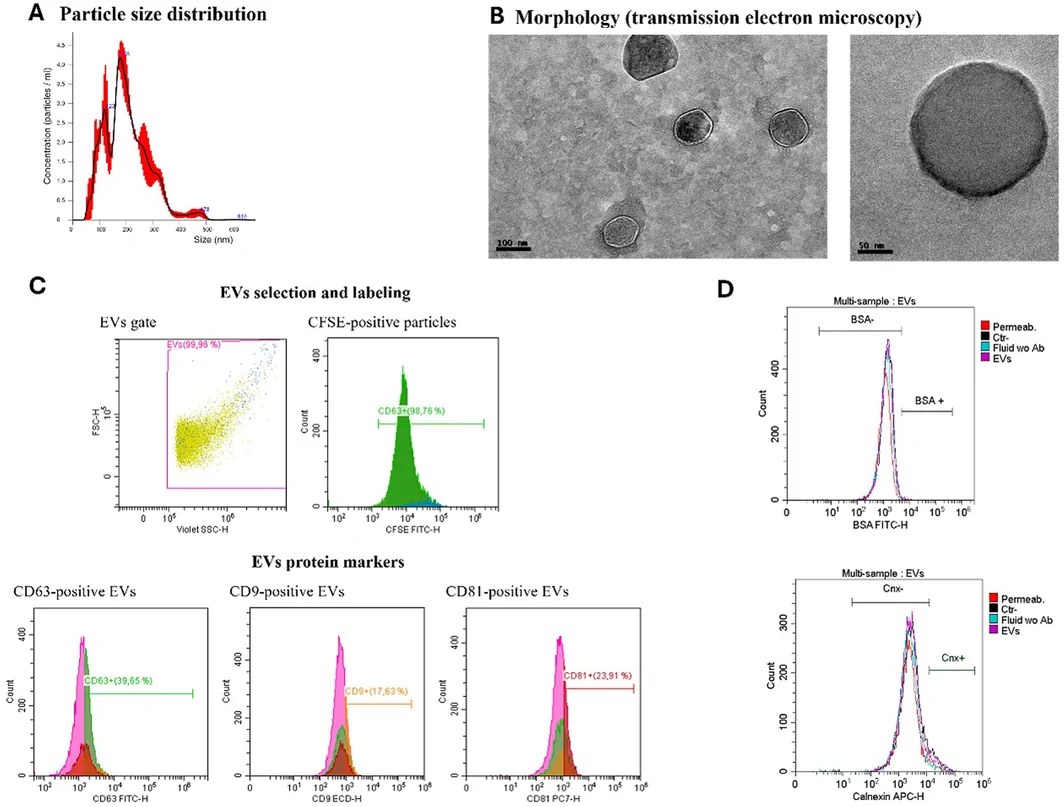
Characterization of extracellular vesicles (EVs) by (A) nanoparticle tracking analysis (NTA), (B) transmission electron microscopy (TEM), and flow cytometry showing (C) the expression of positive EV markers and (D) the absence of negative contamination markers.

### 3.1 ESS‐EVs as Carriers of Key Factors for Sperm Physiology

To test that ESS‐EVs may contribute to the delivery of key molecular signals to spermatozoa, we characterized the proteome of ESS‐EVs and compared it with a signature of 280 proteins previously reported to be upregulated in ram sperm incubated with ESS (Chen et al. 2024). Notably, 76 of these proteins (27.1%) were identified within the ESS‐EVs cargo (Figure 3A). Functional enrichment analysis linked these shared proteins to key processes for sperm function (Table 1), including ATP metabolism and sperm–zona pellucida interaction (Figure 3B). Hypergeometric test confirmed that this overlap was highly significant (*p* = 4.3 × 10^−7^), indicating this is a non‐random enrichment of proteins with function at fertilization within ESS‐EVs.

**FIGURE 3:**
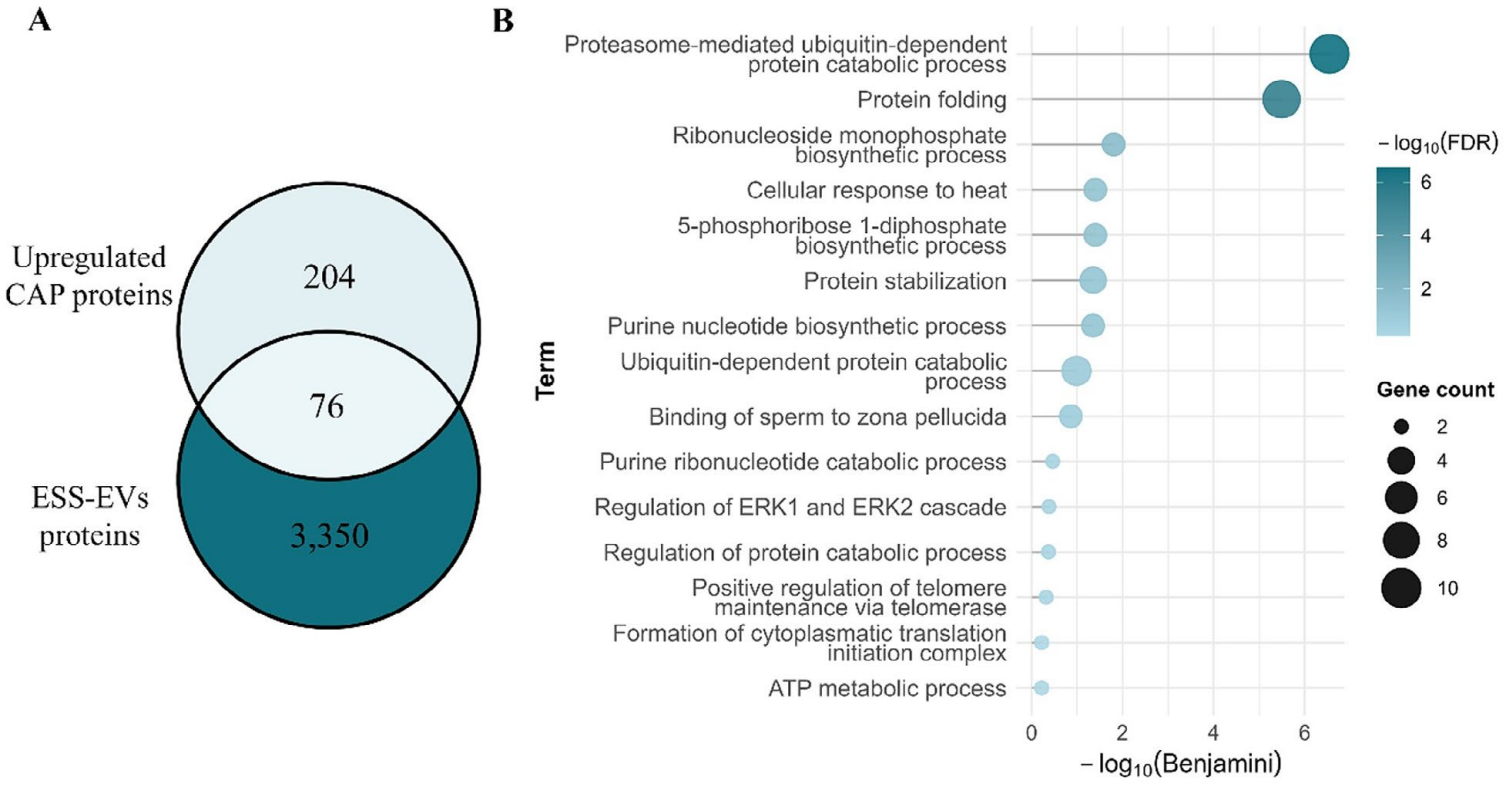
(A) Venn diagram of proteins shared between estrous sheep serum extracellular vesicles (ESS‐EVs) and the capacitation signature (CAP), (B) functional enrichment analysis of these shared proteins.

**TABLE 1:** Functional enrichment analysis of proteins shared between estrous sheep serum extracellular vesicles (ESS‐EVs) and the capacitation signature.

Biological process term	Proteins	*p*	Benjamini
Proteasome‐mediated ubiquitin‐dependent protein catabolic process	10	1.49e‐9	2.85e‐7
Protein folding	9	3.38e‐8	3.22e‐6
Ribonucleoside monophosphate biosynthetic process	3	2.46e‐4	1.57e‐2
Cellular response to heat	3	9.11e‐4	3.97e‐2
5‐Phosphoribose 1‐diphosphate biosynthetic process	3	1.04e‐3	3.97e‐2
Protein stabilization	4	1.39e‐3	4.43e‐2
Purine nucleotide biosynthetic process	3	1.63e‐3	4.46e‐2
Ubiquitin‐dependent protein catabolic process	5	4.3e‐3	1.03e‐1
Binding of sperm to zona pellucida	3	6.45e‐3	1.37e‐1
Purine ribonucleotide catabolic process	2	1.79e‐2	3.42e‐1
Regulation of ERK1 and ERK2 cascade	2	2.38e‐2	4.13e‐1
Regulation of protein catabolic process	2	2.67e‐2	4.25e‐1
Positive regulation of telomere maintenance via telomerase	2	3.26e‐2	4.78e‐1
ATP metabolic process	2	4.41e‐2	5.99e‐1
Formation of cytoplasmatic translation initiation complex	2	4.70e‐2	5.99e‐1

### 3.2 Functional Impact of ESS‐EVs on Sperm Motility and Mitochondrial Activity

To investigate the effects of ESS‐EVs on sperm physiology, sperm and ESS‐EVs were co‐incubated. These co‐incubated sperm exhibited a motility pattern similar to CAP but differed from NCAP in most of the motility descriptors evaluated. ESS‐EVs and CAP samples showed lower SM, VSL, and LIN than the NCAP samples (*p* < 0.05; Figure 4A). In contrast, no differences were found among samples for ALH (*p* > 0.05; Figure 4A). Consistently, mitochondrial activity remained unchanged across the three experimental groups (*p* > 0.05; Figure 4B).

**FIGURE 4:**
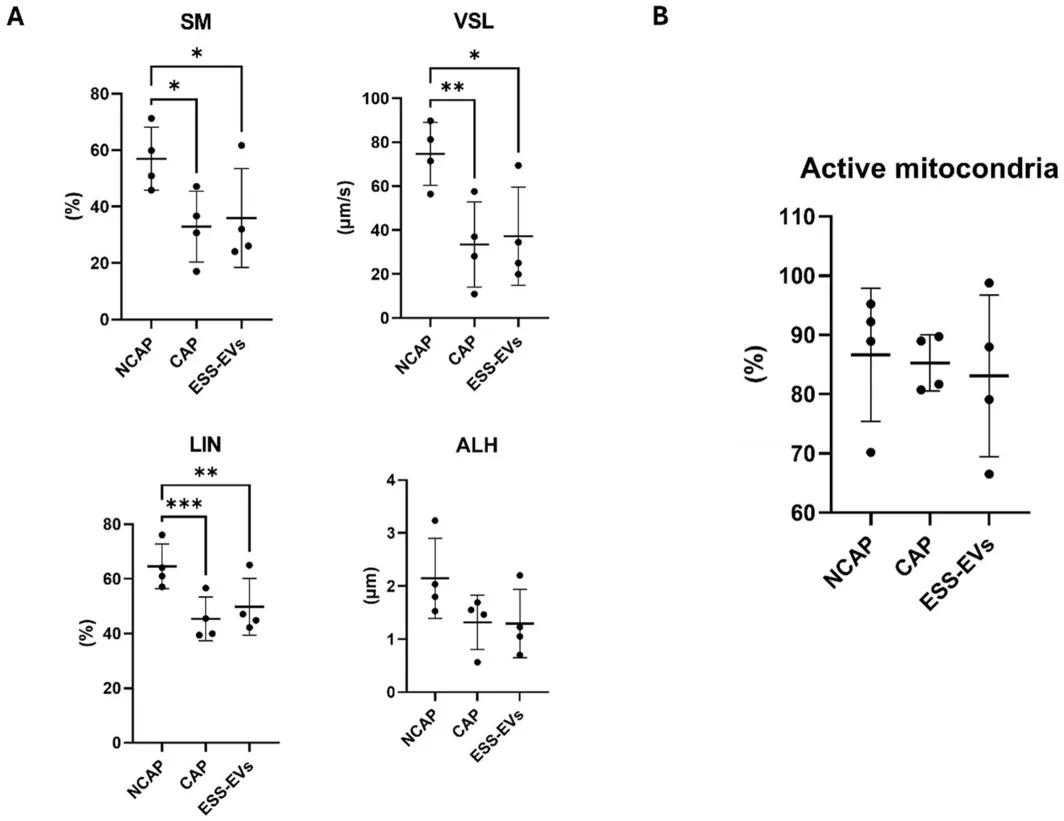
Impact of estrous sheep serum extracellular vesicles (ESS‐EVs) on sperm kinetics and mitochondrial activity compared with positive and negative controls obtained by adding ESS (2%; CAP) or not (NCAP) to a synthetic oviductal fluid. (A) Motility descriptors: Sperm motility (SM); straight line velocity (VSL); linearity (LIN); amplitude of lateral head displacement (ALH). (B) Percentage of viable spermatozoa with active mitochondrial activity. Mean ± SD are shown; (*p < 0.05; **p < 0.01; ***p < 0.001).

## 4 Discussion

The incubation of sperm with ESS results in the upregulation of 280 proteins (Chen et al. 2024), even though these cells are transcriptionally and translationally inactive. This observation strongly suggests that many of these proteins must be acquired from the extracellular environment. However, the limited stability of freely dissolved proteins, together with the evidence that sperm interact with EVs and incorporate their cargo (Picazo‐Córdoba et al. 2024), points to ESS‐EVs as an additional mechanism for protein delivery. Consistent with this possibility, 27.1% of the proteins reported to be upregulated in spermatozoa incubated with ESS were identified within the ESS‐EVs proteome in our study, supporting the idea that these vesicles may act as molecular conveyors involved in the process that prepare sperm for fertilization, as previously proposed (Almiñana et al. 2017). Nevertheless, proteins are unlikely to be the only bioactive components involved, as EV cargo also comprises lipids, miRNAs, and other regulatory molecules that may contribute to the modulation of sperm physiology during capacitation. This proteomic overlap is unlikely to be random, as supported by a significant hypergeometric test indicating genuine enrichment of these factors. These findings strengthen the hypothesis that ESS‐EVs may supply key proteins required for sperm to fertilize, including energy metabolism and zona pellucida binding.

ESS‐EV‐sperm co‐incubation reduced SM to a similar extent as ESS itself, with sperm displaying also lower velocity and linearity. This is consistent with reports showing that EVs from different regions of the woman reproductive tract can modulate sperm motility (Fasoli et al. 2025). However, unlike our findings, those EV populations enhanced motility, highlighting species‐specific functional differences. This reduction in sperm motility was independent of mitochondrial performance, which remained stable across treatments. The absence of changes in mitochondrial activity is noteworthy, as proteins related to ATP metabolism were previously found to be upregulated in sperm after incubation with ESS (Chen et al. 2024), a process that may be partly mediated by its EVs, as suggested herein. However, other EV cargoes, including lipids and miRNAs, may also contribute to the observed effects. Although these proteins could be linked to energy metabolism, their presence did not translate into detectable alterations in mitochondrial activity under the conditions tested. Future studies may clarify whether these proteins contribute to other ATP‐related metabolic pathways supporting the sperm energetic demands associated with fertilization.

## Author Contributions

Conceptualization: María Iniesta‐Cuerda and Ana Josefa Soler; methodology: Clara Vidal‐Garcia; investigation: Clara Vidal‐Garcia, Marta Neila‐Montero, Eva Díaz‐Hernández, Paula Bóveda, Carmen María Picazo‐Córdoba, Vidal Montoro, Olga García‐Álvarez, José Julián Garde, Ana Josefa Soler, María Iniesta‐Cuerda; writing – original draft preparation: Clara Vidal‐Garcia and María Iniesta‐Cuerda; writing‐review and editing: Ana Josefa Soler and José Julián Garde. All authors have read and agreed to the published version of the manuscript.

## Funding

This study was supported by the Ministry of Science, Innovation and Universities https://www.ciencia.gob.es/ca/ (PID2021‐126988OB‐100).

## Conflicts of Interest

The authors declare no conflicts of interest.

## Supporting information


**Table S1:** Differentially abundant proteins identified between non‐capacitated (NCAP) and capacitated (CAP) sheep spermatozoa, adapted from Chen et al. (2024). Adapted from Table S3 published by Chen et al. (2024). The original dataset was simplified by retaining only the information relevant to the comparison performed in this study.

## Data Availability

The data that support the findings of this study are available from the corresponding author upon reasonable request.
