# Supplementary material for: Estrous Sheep Serum‐Derived Extracellular Vesicles Deliver Proteins That Modulate Sperm Motility in Sheep
Source: Reprod Domest Anim. 2026 Sep 30;61(Suppl 3):e70293. doi: 10.1111/rda.70293 (PMC13627753; doi:10.1111/rda.70293)
Supplement: Supplementary file 1 [file RDA-61-e70293-s001.docx]

**Table S1.** Differentially abundant proteins identified between non-capacitated (NCAP) and capacitated (CAP) sheep spermatozoa, adapted from Chen et al. (2024). Adapted from Supplementary Table S3 published by Chen et al. (2024). The original dataset was simplified by retaining only the information relevant to the comparison performed in this study.

| **Protein accession** | **Regulated Type** | **Ratio** | **P value** |
| --- | --- | --- | --- |
| W5PJB6 | Up | 1,602 | 0,00038 |
| W5PTW5 | Up | 1,933 | 0,00176 |
| W5P645 | Up | 1,901 | 1,5E-06 |
| W5Q5R8 | Up | 1,547 | 0,00048 |
| W5PQ34 | Up | 1,631 | 0,00388 |
| W5PFJ0 | Up | 1,529 | 0,0031 |
| W5P3I5 | Up | 1,542 | 0,00012 |
| W5P2V3 | Up | 1,909 | 0,00042 |
| W5Q2D8 | Up | 1,699 | 0,00012 |
| W5QE19 | Up | 1,575 | 0,00026 |
| W5QDG0 | Up | 1,61 | 0,00044 |
| W5QBE9 | Up | 1,711 | 0,00048 |
| W5P228 | Up | 1,887 | 8,7E-07 |
| Q2TCH3 | Up | 1,944 | 2,8E-06 |
| W5PHB8 | Up | 1,567 | 0,00394 |
| W5PIL6 | Up | 1,518 | 0,00066 |
| W5PIJ2 | Up | 1,514 | 2,4E-06 |
| W5NWN2 | Up | 1,762 | 2,2E-05 |
| W5P610 | Up | 2,191 | 0,0139 |
| W5QH42 | Up | 1,632 | 4,4E-05 |
| W5PC09 | Up | 1,515 | 2,3E-05 |
| W5PWF2 | Up | 1,614 | 1,5E-05 |
| W5Q6E0 | Up | 1,978 | 0,00022 |
| W5Q785 | Up | 1,677 | 0,00024 |
| W5P9M2 | Up | 1,569 | 0,00208 |
| W5QCP6 | Up | 1,658 | 0,00012 |
| W5P3E8 | Up | 1,543 | 0,00242 |
| W5PC18 | Up | 1,813 | 1,6E-05 |
| W5P8Z3 | Up | 1,87 | 2E-05 |
| W5Q7C7 | Up | 2,089 | 0,00086 |
| W5Q701 | Up | 1,812 | 0,00012 |
| W5PFU1 | Up | 1,624 | 2E-05 |
| W5P316 | Up | 1,681 | 3,8E-05 |
| W5PP87 | Up | 1,896 | 0,00072 |
| W5QGT1 | Up | 1,635 | 0,0002 |
| W5QEP5 | Up | 1,662 | 0,00118 |
| W5NRA2 | Up | 1,916 | 0,00022 |
| W5Q9B2 | Up | 2,076 | 0,0002 |
| W5PHW0 | Up | 1,816 | 0,0001 |
| W5QD30 | Up | 1,545 | 0,0002 |
| W5P8B4 | Up | 1,941 | 9,5E-05 |
| W5PPS6 | Up | 1,922 | 0,00044 |
| W5QCY9 | Up | 2,369 | 3,9E-05 |
| W5P765 | Up | 1,593 | 1,6E-05 |
| W5P8T5 | Up | 1,811 | 0,00158 |
| W5NZ76 | Up | 5,522 | 0,0001 |
| W5PC82 | Up | 2,067 | 1,7E-05 |
| W5PBH2 | Up | 1,612 | 5,6E-05 |
| W5PYG7 | Up | 1,502 | 4,1E-06 |
| W5QAH0 | Up | 1,574 | 0,00034 |
| W5QCW3 | Up | 1,547 | 7,5E-05 |
| W5NQ14 | Up | 1,535 | 0,00702 |
| W5PVU5 | Up | 2,03 | 0,00568 |
| W5P363 | Up | 2,516 | 0,0003 |
| W5Q038 | Up | 1,636 | 0,00102 |
| W5PE22 | Up | 1,682 | 0,00214 |
| A7UHZ2 | Up | 1,515 | 0,00142 |
| W5NT10 | Up | 1,572 | 0,00012 |
| W5QIT0 | Up | 2,557 | 0,00172 |
| W5P0V5 | Up | 1,864 | 7,8E-05 |
| W5PRP1 | Up | 1,742 | 7,6E-05 |
| W5PTU7 | Up | 1,802 | 0,00022 |
| W5PJC2 | Up | 1,685 | 0,00204 |
| W5PBK1 | Up | 1,71 | 0,00056 |
| W5NUG3 | Up | 1,513 | 0,00052 |
| W5NSV2 | Up | 1,982 | 0,0002 |
| W5Q420 | Up | 2,486 | 5,6E-05 |
| W5PVL5 | Up | 1,781 | 0,00014 |
| W5P8K0 | Up | 1,907 | 0,00192 |
| W5P6F9 | Up | 1,697 | 4,3E-05 |
| W5PQG6 | Up | 1,965 | 2,7E-07 |
| W5QCD6 | Up | 1,855 | 0,005 |
| W5P9U4 | Up | 1,695 | 7,7E-05 |
| W5PNZ5 | Up | 1,967 | 7,6E-05 |
| W5Q6N7 | Up | 1,652 | 4E-05 |
| W5Q0A8 | Up | 1,77 | 0,00134 |
| W5QBD7 | Up | 1,623 | 5,5E-05 |
| W5P4M2 | Up | 1,614 | 8,4E-05 |
| W5QFN9 | Up | 2,015 | 8,3E-05 |
| W5PV74 | Up | 1,778 | 0,00024 |
| W5PD43 | Up | 1,735 | 0,01564 |
| W5PIC3 | Up | 1,552 | 0,00016 |
| W5PPD3 | Up | 1,981 | 3,6E-05 |
| W5PL69 | Up | 1,892 | 0,00018 |
| W5QHM6 | Up | 1,511 | 0,00748 |
| W5PMS2 | Up | 1,622 | 1,6E-05 |
| W5PUH7 | Up | 1,962 | 8,4E-05 |
| W5Q8A7 | Up | 1,608 | 0,00024 |
| W5P9R3 | Up | 1,673 | 3,3E-06 |
| W5PSF8 | Up | 1,557 | 0,00028 |
| W5NZZ3 | Up | 1,622 | 0,00316 |
| A8DR93 | Up | 1,814 | 6,2E-07 |
| W5PZ65 | Up | 1,613 | 0,00046 |
| W5Q3B4 | Up | 1,541 | 0,00064 |
| W5Q694 | Up | 1,718 | 0,00032 |
| W5QEY8 | Up | 1,546 | 0,00058 |
| W5NVQ4 | Up | 1,813 | 2,3E-05 |
| W5NW97 | Up | 1,969 | 0,00318 |
| W5NZY8 | Up | 2,245 | 0,0002 |
| W5P375 | Up | 1,717 | 1,7E-06 |
| W5PSF3 | Up | 1,524 | 0,00016 |
| W5PYM5 | Up | 1,864 | 1,6E-05 |
| W5Q6H1 | Up | 1,821 | 0,0236 |
| W5PXX7 | Up | 1,702 | 1,7E-05 |
| W5PNX6 | Up | 1,81 | 0,00026 |
| W5PNT5 | Up | 1,57 | 0,00032 |
| W5NPE2 | Up | 6,828 | 2E-06 |
| W5Q922 | Up | 1,676 | 3,8E-05 |
| W5PRJ4 | Up | 1,595 | 0,00096 |
| W5NYH2 | Up | 1,532 | 3,2E-06 |
| W5PJX1 | Up | 2,182 | 6,7E-07 |
| W5PFC9 | Up | 4,232 | 1,1E-06 |
| W5Q4D9 | Up | 1,729 | 9,9E-05 |
| W5PE35 | Up | 3,399 | 1,6E-06 |
| W5QHL1 | Up | 4,97 | 2E-05 |
| W5Q7P4 | Up | 2,132 | 0,00012 |
| W5Q8X9 | Up | 1,542 | 0,0002 |
| W5Q5L6 | Up | 1,98 | 0,0001 |
| W5PHM0 | Up | 1,709 | 0,00012 |
| P14639 | Up | 7,892 | 0,00102 |
| W5PD85 | Up | 1,504 | 0,00036 |
| W5PG90 | Up | 1,815 | 0,00262 |
| W5QGS5 | Up | 1,967 | 0,00066 |
| W5P1I7 | Up | 1,541 | 0,0054 |
| W5PGE9 | Up | 1,615 | 0,00044 |
| W5Q9K1 | Up | 1,876 | 0,00276 |
| W5Q0Z2 | Up | 4,273 | 2,2E-05 |
| W5PTS4 | Up | 1,676 | 0,00026 |
| W5PQD0 | Up | 3,266 | 0,00014 |
| W5NUH6 | Up | 1,592 | 4E-05 |
| W5P3C6 | Up | 2,397 | 2E-05 |
| W5PSZ8 | Up | 1,66 | 0,00044 |
| W5PSK0 | Up | 1,506 | 0,00022 |
| W5P9V5 | Up | 2,669 | 8,1E-05 |
| W5QAX4 | Up | 1,557 | 0,00202 |
| W5PFF7 | Up | 2,409 | 6,3E-05 |
| W5QHU9 | Up | 3,305 | 3,9E-05 |
| W5PRU5 | Up | 4,855 | 3,6E-06 |
| W5PST9 | Up | 1,534 | 0,00014 |
| W5NU23 | Up | 1,523 | 0,00034 |
| W5QHZ5 | Up | 2,783 | 0,00034 |
| W5NQ55 | Up | 3,538 | 4E-05 |
| P0C276 | Up | 2,719 | 2,2E-05 |
| W5QI29 | Up | 4,221 | 8E-05 |
| W5NTL9 | Up | 1,571 | 0,00114 |
| W5NSH8 | Up | 5,301 | 3,2E-06 |
| W5PGP1 | Up | 4,821 | 3,6E-05 |
| P56283 | Up | 3,355 | 4,4E-05 |
| W5PSQ7 | Up | 2,788 | 2,3E-05 |
| W5P861 | Up | 4,919 | 1,7E-05 |
| W5QHX9 | Up | 1,61 | 0,00036 |
| W5PBI1 | Up | 3,866 | 2,2E-05 |
| W5PJ13 | Up | 1,555 | 0,00354 |
| W5PG72 | Up | 2,987 | 0,00052 |
| W5PYJ8 | Up | 2,011 | 4,2E-07 |
| W5P8Z1 | Up | 3,077 | 5,5E-05 |
| W5NS55 | Up | 3,566 | 2,8E-06 |
| W5Q691 | Up | 1,589 | 0,04476 |
| W5P7X5 | Up | 7,052 | 2,3E-05 |
| W5P887 | Up | 2,063 | 0,00604 |
| W5PZD0 | Up | 6,098 | 4,7E-06 |
| W5PFP1 | Up | 2,939 | 1,1E-07 |
| W5QH54 | Up | 4,47 | 1E-06 |
| W5NZA0 | Up | 2,145 | 0,0008 |
| W5QJB1 | Up | 2,774 | 4,9E-07 |
| W5PSL3 | Up | 2,697 | 4,2E-06 |
| W5NQA4 | Up | 2,005 | 9,8E-05 |
| W5Q0G6 | Up | 1,538 | 3,8E-05 |
| H9TN93 | Up | 3,02 | 1,7E-05 |
| W5PD71 | Up | 2,828 | 3,6E-05 |
| W5PR14 | Up | 3,682 | 0,00022 |
| W5Q9A2 | Up | 3,858 | 2,5E-05 |
| W5PEB3 | Up | 3,854 | 3E-07 |
| W5PVG2 | Up | 5,134 | 2,2E-05 |
| W5QEP3 | Up | 2,801 | 1,6E-05 |
| W5NZB8 | Up | 2,013 | 7,7E-05 |
| W5PAK0 | Up | 2,339 | 4,5E-05 |
| W5PCT0 | Up | 2,386 | 0,00016 |
| W5Q5N9 | Up | 1,813 | 2E-05 |
| W5PP11 | Up | 1,997 | 0,00178 |
| W5QED7 | Up | 2,136 | 7,9E-05 |
| W5P689 | Up | 1,797 | 0,0001 |
| W5PZA6 | Up | 2,142 | 0,00232 |
| W5Q9H0 | Up | 4,056 | 2,3E-05 |
| W5PH25 | Up | 1,62 | 0,0002 |
| W5PBA2 | Up | 1,96 | 0,0023 |
| W5NZR9 | Up | 1,723 | 1,7E-06 |
| W5PX87 | Up | 1,643 | 0,0153 |
| W5NYE6 | Up | 3,295 | 4,1E-05 |
| W5P508 | Up | 1,576 | 3,5E-05 |
| W5NZ92 | Up | 1,991 | 0,00124 |
| W5PUS0 | Up | 1,512 | 0,00022 |
| W5QEV8 | Up | 1,684 | 0,00074 |
| W5PT81 | Up | 1,816 | 4,5E-05 |
| W5NRL8 | Up | 1,707 | 0,00243 |
| W5PYQ5 | Up | 1,77 | 7,9E-05 |
| W5Q149 | Up | 2,13 | 7,2E-07 |
| W5NUW7 | Up | 1,527 | 0,00066 |
| W5QIC3 | Up | 1,58 | 0,001 |
| W5NT90 | Up | 1,574 | 0,00054 |
| W5P2F6 | Up | 1,635 | 0,00038 |
| W5P7T9 | Up | 2,033 | 0,00016 |
| W5PVT6 | Up | 1,85 | 2,7E-06 |
| W5PXE3 | Up | 1,588 | 0,00056 |
| W5Q625 | Up | 1,832 | 8E-05 |
| W5QFN1 | Up | 1,7 | 0,00066 |
| W5PGV7 | Up | 1,743 | 0,00014 |
| W5PG92 | Up | 2,039 | 0,0001 |
| W5Q4E7 | Up | 1,749 | 0,00198 |
| W5PUM8 | Up | 1,678 | 1,6E-05 |
| W5QDU4 | Up | 1,574 | 0,00028 |
| W5PVN4 | Up | 1,928 | 0,0001 |
| W5NSS1 | Up | 1,805 | 1,9E-05 |
| W5P3X6 | Up | 2,11 | 7,7E-05 |
| W5Q8I4 | Up | 2,262 | 1,3E-06 |
| W5PB94 | Up | 2,469 | 0,00018 |
| W5PTA5 | Up | 1,515 | 0,00066 |
| W5PLZ0 | Up | 1,792 | 2,1E-05 |
| W5P0Z7 | Up | 1,542 | 0,00554 |
| W5Q1M0 | Up | 3,614 | 3,5E-06 |
| W5PDL8 | Up | 1,829 | 0,00092 |
| W5PE27 | Up | 1,584 | 0,0018 |
| W5PJ55 | Up | 1,678 | 0,00088 |
| W5Q4X4 | Up | 1,678 | 0,00036 |
| W5Q0L1 | Up | 1,52 | 0,01342 |
| W5P0T8 | Up | 1,761 | 0,0002 |
| W5P9K6 | Up | 1,938 | 2,5E-07 |
| W5Q9H2 | Up | 1,623 | 0,00012 |
| W5QEE5 | Up | 1,787 | 0,0007 |
| W5QBM5 | Up | 1,712 | 0,00832 |
| W5PTV7 | Up | 2,547 | 9E-07 |
| W5PJT5 | Up | 2,192 | 0,00094 |
| W5PSJ3 | Up | 2,479 | 1,1E-07 |
| W5PGV9 | Up | 1,634 | 0,00188 |
| W5NZ87 | Up | 2,964 | 0,0004 |
| W5Q472 | Up | 1,597 | 0,0046 |
| W5P3S1 | Up | 2,413 | 5,5E-05 |
| W5PXF6 | Up | 2,605 | 0,0013 |
| W5PT79 | Up | 2,044 | 2E-05 |
| W5Q7U9 | Up | 2,241 | 0,0001 |
| W5NYA8 | Up | 1,63 | 3,1E-06 |
| W5PAJ3 | Up | 4,57 | 1,5E-06 |
| W5PGH7 | Up | 1,803 | 0,0005 |
| W5QHD8 | Up | 1,663 | 0,00026 |
| W5Q1P6 | Up | 1,576 | 8,5E-05 |
| W5PRG8 | Up | 3,243 | 2,1E-06 |
| W5PXJ0 | Up | 1,612 | 0,00024 |
| W5P2P7 | Up | 2,117 | 3,7E-05 |
| W5NZL3 | Up | 2,472 | 0,00014 |
| W5Q2E3 | Up | 1,793 | 3,2E-06 |
| W5NZ10 | Up | 1,568 | 0,00036 |
| W5QIT9 | Up | 1,536 | 0,00178 |
| W5QDM2 | Up | 1,62 | 0,0043 |
| W5QAA1 | Up | 2,056 | 0,00072 |
| W5PIE9 | Up | 1,786 | 0,0012 |
| W5P707 | Up | 1,647 | 0,00046 |
| W5PB50 | Up | 1,6 | 0,0007 |
| W5PYX5 | Up | 2,048 | 0,00522 |
| W5QDN6 | Up | 1,54 | 0,00052 |
| W5PD35 | Up | 1,694 | 0,0014 |
| W5NZ70 | Up | 1,737 | 0,00046 |
| W5QIL2 | Up | 1,667 | 0,00024 |
| W5NUE4 | Up | 5,458 | 2,2E-05 |
| W5NPY4 | Up | 2,752 | 7,7E-05 |
| W5P1C2 | Up | 4,335 | 2,2E-06 |
| W5Q4L4 | Up | 1,605 | 0,00616 |
| W5PS94 | Up | 3,632 | 2,6E-06 |
| W5PBG8 | Up | 2,75 | 2,4E-06 |
| W5QGM9 | Up | 2,531 | 2,4E-05 |
| W5PXE0 | Up | 1,543 | 0,0016 |
| W5P0B7 | Up | 2,739 | 1,8E-06 |
| W5PUV3 | Up | 2,408 | 9,6E-05 |
| W5NSM1 | Up | 1,998 | 0,00212 |
| W5PS88 | Up | 1,625 | 0,00028 |
| W5Q0G8 | Up | 1,562 | 0,0184 |
| W5QGX6 | Up | 1,627 | 0,00064 |
| W5QI69 | Up | 1,643 | 4,4E-05 |
| W5P1T4 | Up | 1,517 | 0,01114 |
| W5NPI3 | Up | 1,697 | 0,01104 |
| W5QB42 | Up | 5,125 | 0,00437 |
| W5NQT7 | Down | 0,652 | 0,00114 |
| W5PIB4 | Down | 0,666 | 0,00032 |
| W5P9X2 | Down | 0,657 | 0,00058 |
| W5P066 | Down | 0,623 | 0,0046 |
| W5PDC1 | Down | 0,437 | 1,8E-06 |
| W5P9A0 | Down | 0,58 | 0,01942 |
| W5PX31 | Down | 0,452 | 5,7E-05 |
| W5NZM0 | Down | 0,634 | 6,1E-05 |
| W5P718 | Down | 0,598 | 0,00024 |
| W5Q6V1 | Down | 0,603 | 0,00766 |
| W5NZ60 | Down | 0,637 | 0,00032 |
| P02075 | Down | 0,396 | 6E-07 |
| W5QDB7 | Down | 0,502 | 3,9E-05 |
| W5QBX3 | Down | 0,654 | 0,01038 |
| W5P1S3 | Down | 0,6 | 7,8E-05 |
| W5PP37 | Down | 0,612 | 0,00525 |
| W5P428 | Down | 0,481 | 0,00012 |
| W5NYG6 | Down | 0,631 | 0,02414 |
| W5P657 | Down | 0,483 | 0,00328 |
| W5QD72 | Down | 0,632 | 0,02342 |
| B2MVX2 | Down | 0,612 | 0,00258 |
| W5NX61 | Down | 0,543 | 5,8E-05 |
| W5Q3H2 | Down | 0,376 | 3,5E-06 |
| W5NTK4 | Down | 0,636 | 0,00012 |
| W5NSC3 | Down | 0,568 | 4,1E-06 |
| W5Q6T4 | Down | 0,634 | 7,8E-05 |
| W5P953 | Down | 0,324 | 2,1E-05 |
| W5NQB6 | Down | 0,427 | 3,7E-06 |
| W5QJ78 | Down | 0,548 | 0,02582 |
| W5NSD8 | Down | 0,635 | 3,7E-05 |
| W5PN67 | Down | 0,398 | 0,0027 |
| W5NT58 | Down | 0,548 | 1,7E-05 |
| W5NV01 | Down | 0,661 | 0,0315 |
| W5PK35 | Down | 0,614 | 0,00184 |
| W5Q257 | Down | 0,649 | 2,5E-05 |
| W5Q157 | Down | 0,655 | 0,01918 |
| W5P9D5 | Down | 0,53 | 0,0078 |
| W5NZF1 | Down | 0,52 | 0,00086 |
| W5QG11 | Down | 0,549 | 4,9E-07 |
| W5PKH1 | Down | 0,316 | 5,7E-05 |
| W5PTW2 | Down | 0,428 | 4E-05 |
| W5NU53 | Down | 0,618 | 0,02738 |
| W5PG49 | Down | 0,572 | 0,00052 |
| W5PJK2 | Down | 0,444 | 0,00018 |
| W5QDL6 | Down | 0,653 | 1,2E-06 |
| W5PFD6 | Down | 0,55 | 0,00082 |
| W5QI90 | Down | 0,659 | 0,00024 |
| W5Q4Y3 | Down | 0,637 | 0,00508 |
| W5PRH4 | Down | 0,607 | 0,00026 |
| W5PW99 | Down | 0,465 | 0,0177 |
| W5NSF8 | Down | 0,652 | 0,00588 |
| W5PM77 | Down | 0,454 | 0,00102 |
| W5P7F4 | Down | 0,637 | 0,0002 |
| W5NZL1 | Down | 0,657 | 0,01928 |
| W5QHI0 | Down | 0,657 | 0,00816 |
| W5PUP9 | Down | 0,658 | 0,00172 |
| W5NTL3 | Down | 0,615 | 4,3E-05 |
| W5P4B3 | Down | 0,642 | 0,00016 |
| W5PE07 | Down | 0,521 | 0,00028 |
| W5PTU5 | Down | 0,535 | 0,00064 |
| W5PQW1 | Down | 0,652 | 0,00252 |
| W5QFB9 | Down | 0,617 | 0,00016 |
| W5Q3Y4 | Down | 0,478 | 0,00474 |
| W5Q770 | Down | 0,59 | 0,0002 |
| W5PI81 | Down | 0,55 | 9,6E-05 |
| W5PHH6 | Down | 0,532 | 0,0035 |
| W5PBF9 | Down | 0,499 | 0,00042 |
| W5QEU1 | Down | 0,66 | 0,00216 |
